# Surveys on the prevalence of pediatric asthma in Japan: A comparison between the 1982, 1992, 2002, 2012, and 2022 surveys conducted in the same region using the same methodology (WJSAAC PhaseⅠ∼Ⅴ)

**DOI:** 10.1016/j.waojou.2025.101052

**Published:** 2025-04-23

**Authors:** Sankei Nishima, Junichiro Tezuka, Hiroshi Odajima, Hiroshi Matsuzaki, Satoshi Honjo, Kunitaka Ota, Yuji Gohda, Kazuyoshi Koga, Keijiro Tsuda, Yoshiaki Kanaya, Shuich Yamamoto, Shinji Aratake, Tatsuo Koga, Ayako Sakamoto, Yasushi Shimada, Kasumi Tashiro, Kazunari Fukahori, Shinpei Sunagawa, Hiroki Yamamoto, Hiroyuki Matsuzaki, Toru Nakamura, Kiyoshi Nishikawa, Kazumi Hiraba, Tokihiko Fujino, Takashi Fujiwara, Maeda Yoshiteru, Shigetaka Matsumoto, Chiho Tatsumoto, Zenji Miyazato

**Affiliations:** a Department of Pediatrics, NHO Fukuoka National Hospital, Fukuoka, Japan; b Department of Allergy and Pulmonology, Fukuoka Children's Hospital, Fukuoka, Japan; c Yanagawa Institute for Developmental Disabilities, Yanagawa, Japan; d Department of Pediatrics, Konan Medical Center, Hyogo, Japan; e Department of Pediatrics, Sasebo Kyosai Hospital, Nagasaki, Japan; f Kitakyushu Medical Association, Fukuoka, Japan; g Oita Medical Association, Oita, Japan; h Department of Pediatrics, NHO Higashisaga Hospital, Saga, Japan; i Department of Pediatrics, NHO Ibusuki Medical Center, Kagoshima, Japan; j Omuta Medical Association, Fukuoka, Japan; k Department of Pediatrics, Nagasaki Harbor Medical Center, Nagasaki, Japan; l Amakusa Medical Association, Kumamoto, Japan; m Department of Pediatrics, JCHO Isahaya General Hospital, Nagasaki, Japan; n Oomura Medical Association, Nagasaki, Japan; o Sanyo Onoda Medical Association, Yamaguchi, Japan; p Department of Pediatrics, Miyazaki Seikyo Hospital, Miyazaki, Japan; q Shimonoseki Medical Association, Yamaguchi, Japan; r Department of Pediatrics, Kagoshima Seikyou General Hospital, Kagoshima, Japan; s Kagawa Medical Association, Kagawa, Japan; t Department of Pediatrics, Shoushinkai Mito Hospital, Fukuoka, Japan; u Tamana Medical Association, Kumamoto, Japan; v Beppu Medical Association, Oita, Japan; w Kagoshima City Medical Association, Kagoshima, Japan; x Department of Pediatrics, Nakagami Hospital, Okinawa, Japan

**Keywords:** Prevalence, Asthma, Wheeze, Allergic disease, COVID-19

## Abstract

**Objectives:**

To investigate the trends in the prevalence of pediatric asthma in the western part of Japan over 40 years, using data from surveys conducted every 10 years from 1982 to 2022.

**Design:**

A series of school-based epidemiological surveys conducted every 10 years.

**Setting:**

Elementary schools in 11 prefectures in the western part of Japan.

**Participants:**

Children aged 6–12 years. Surveys included 55,388 children in 1982, 46,718 in 1992, 36,228 in 2002, 33,902 in 2012, and 30,024 in 2022.

**Main outcome measures:**

Prevalence of asthma, wheeze, and other allergic diseases such as allergic rhinitis, atopic dermatitis, and food allergies.

**Results:**

The prevalence of asthma increased from 1982 to 2002 (boys: 3.8%–8.1%; girls: 2.5%–5.0%) and then decreased in 2012 and 2022 (boys: 6.0%–3.2%; girls: 3.5%–2.1%). The prevalence of wheeze followed a similar trend. Allergic rhinitis was the most common comorbidity, affecting 53–58% of children from 1992 onwards. Other comorbidities included atopic dermatitis, allergic conjunctivitis, and pollinosis. The prevalence of asthma was higher in urban areas than in rural areas in the early surveys, but this difference disappeared in later surveys.

**Conclusions:**

The prevalence of pediatric asthma in western Japan increased until 2002 and then decreased in subsequent surveys. Changes in environmental factors and public health measures, including those related to the COVID-19 pandemic, may have influenced these trends. Further research is needed to understand the long-term effects of these factors on asthma prevalence.

## Introduction

The increase in allergic diseases has been a global concern, leading to the establishment of the International Study of Asthma and Allergies in Childhood (ISAAC) to understand the prevalence and causes. Since 1995, Phase I trials have been conducted,^1^, ^2^, ^3^, ^4^ followed by Phase II trials, and from 2001 to 2002, Phase III trials, with Japan participating in Phases I, III, and parts of Phase II. In western Japan, the first survey on the prevalence of asthma in elementary school children was conducted in 1982, targeting 55,000 children (West Japan Study of Asthma and Allergies in Childhood, WJSAAC-I).^5^

We expanded the survey to include other allergic diseases in subsequent surveys conducted in 1992 (WJSAAC-II), 2002 (WJSAAC-III), 2012 (WJSAAC-IV), and 2022 (WJSAAC-V).^6^, ^7^, ^8^ These surveys showed that the prevalence of asthma, which had been increasing since 1982, peaked in 2002 and then decreased significantly in the surveys conducted in 2012 and 2022. This study reports on the trends observed in these 5 surveys.

## Methods

### Study population

The study population included all elementary school children from grades 1 to 6 in 11 prefectures in western Japan, including all prefectures in Kyushu, Yamaguchi, Hyogo, and Kagawa. The cross-sectional studies were conducted in 1982 (WJSAAC-I), 1992 (WJSAAC-II), 2002 (WJSAAC-III), 2012 (WJSAAC-IV), and 2022 (WJSAAC-V). The response rates were 95.9% in WJSAAC-I, 96.8% in WJSAAC-II, 96.1% in WJSAAC-III, 96.2% in WJSAAC-IV, and 93.0% in WJSAAC-V, with a total of 55,388, 46,718, 36,228, 33,902, and 30,024 children analyzed, respectively (Table 1). This period saw a decline in the pediatric population across Japan.

**Table 1:** Study subjects by year at survey, sex and type of residential area.

Year at survey	No of school	n			Response Rate (%)	Area (%)		
		Male	Female	Total		Urban	Middle	Rural
1982 (WJSAAC-Ⅰ)	70	28036 (50.6%)	27352 (49.4%)	55388	95.9	36.9	57.0	6.1
1992 (WJSAAC-Ⅱ)	79	23574 (50.5%)	23144 (49.5%)	46718	96.8	30.7	64.7	4.6
2002 (WJSAAC-Ⅲ)	81	18264 (50.4%)	17964 (49.6%)	36228	96.1	33.1	62.7	4.2
2012 (WJSAAC-Ⅳ)	81	17217 (50.8%)	16685 (49.2%)	33902	96.2	29.4	66.1	4.5
2022 (WJSAAC-Ⅴ)	76	15250 (50.8%)	14657 (48.8%)	30024	93.0	32.5	62.7	4.8

Age: 6–12 years.

Changes in number of schools were due to reorganization including branching and consolidation.

Area is classified by population density. Urban 1500∼/km^2^, middle 250–1500/km^2^, rural ∼250/km^2^

### Survey instrument

A modified Japanese version of the American Thoracic Society - Division of Lung Diseases (ATS-DLD) questionnaire was used in all 5 surveys. The Japanese version of the ATS-DLD questionnaire was developed by the Japan Public Health Association,^9^ and it has been validated for use in Japanese children.^10^ The definition of asthma remained consistent across the surveys. Participants were considered to have asthma if they met all the following criteria:^9^^,^^11^, ^12^, ^13^, ^14^ (1) experienced wheezing and/or shortness of breath; (2) had 2 or more attacks in the past; (3) diagnosed with asthma by a doctor; (4) wheezing heard during an attack; (5) experienced shortness of breath and wheezing during an attack; and (6) experienced such an attack or received treatment for asthma in the past 2 years. Remission was defined as the absence of asthma symptoms and no treatment for asthma in the past 2 years. Participants who met the following 3 criteria, but did not meet the criteria for asthma or asthma remission were considered to have wheeze: (1) occasional wheezing; (2) wheezing symptoms occurred with a cold; and (3) experienced wheezing twice or more in the past 2 years. The definition criteria for allergic diseases are shown in Supplementary Table S1.

### Study areas

The study areas were categorized into 3 groups based on population density: urban (1500 people/km^2^ or more), middle (250–1500 people/km^2^), and rural (250 people/km^2^ or less). The prevalence of asthma was examined by school grade, prefecture, sex, and the categorized study areas, as well as combinations of these categories.

### Environmental data

The relationships between asthma prevalence and concentrations of nitric oxide (NOx) and suspended particulate matter (SPM) were examined. NOx and SPM levels were measured by the National Institute for Environmental Studies at 27 air quality monitoring stations within 2 km of each elementary school. SOx levels were not examined due to their recent declining trend in Japan.

### Ethics statement

This study was approved by the Institutional Review Board of Fukuoka Children's Hospital (No. 2021-963). It was conducted in accordance with the principles laid out in the Helsinki Declaration and other national regulations and guidelines.

### Statistical analysis

Intercategory differences were analyzed using the chi-squared test. Statistical analyses were performed using SPSS 20.0J for Windows (SPSS Japan, Tokyo, Japan). The risk of having asthma due to a family history of allergic diseases (father, mother, or both) and history of severe cold or respiratory tract infection at 2 years of age or younger was examined by calculating odds ratios (ORs). Missing values were excluded from the analysis. A significance level of p < 0.05 was adopted.

## Results

### Prevalence of asthma

**Fig. 1:**
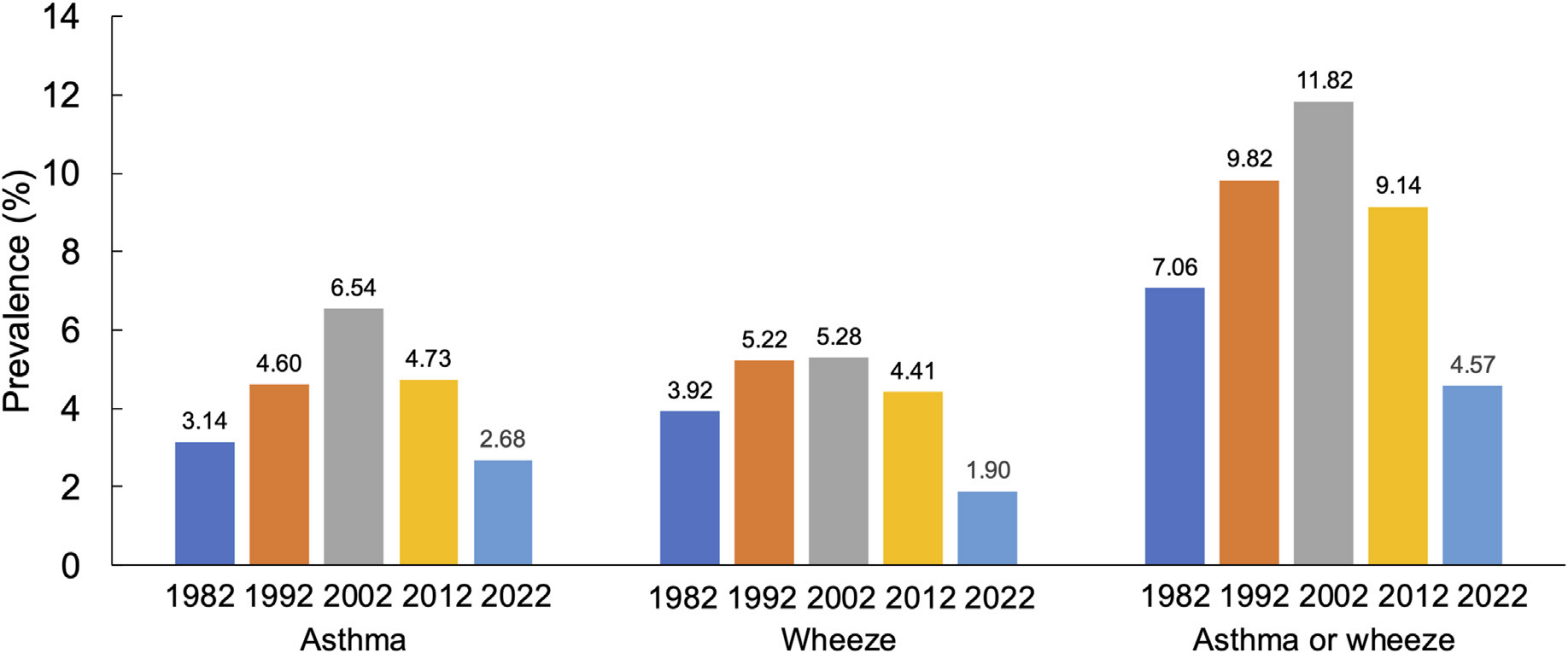
Prevalence of asthma and wheeze in 1982, 1992, 2002, 2012 and 2022

**Fig. 2:**
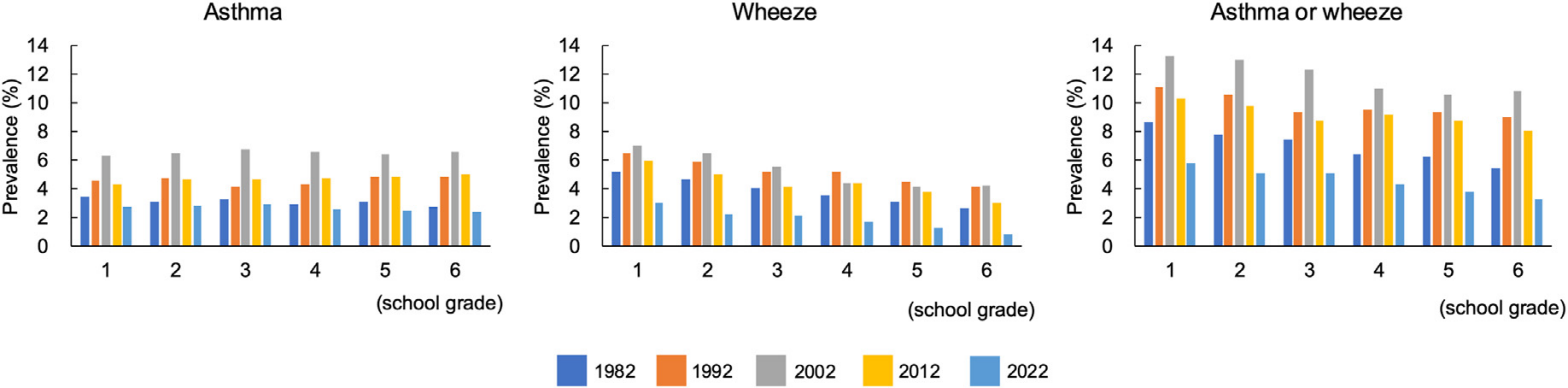
Prevalence of asthma and wheeze in elemental school grade

The number of children surveyed is shown in Table 1. The prevalence of asthma in boys was 3.8% in 1982, 5.6% in 1992, 8.1% in 2002, 6.0% in 2012, and 3.2% in 2022. For girls, the prevalence was 2.5% in 1982, 3.6% in 1992, 5.0% in 2002, 3.5% in 2012, and 2.1% in 2022. Overall, the prevalence were 3.2%, 4.6%, 6.5%, 4.7%, and 2.7%, respectively. Both boys and girls showed the highest prevalence in 2002, with a decrease observed in 2012 and 2022, the lowest being in 2022 (Table S2). The prevalence of asthma, wheeze, and asthma or wheeze increased significantly from Phase I to Phase III (p for trend <0.001), but decreased in 2012 and 2022 (Fig. 1). The trend by school grade is shown in Fig. 2, showing no decrease in prevalence with increasing grade for asthma. The ratio of boys to girls was consistently higher in boys, with ratios of 1.6, 1.6, 1.6, 1.7, and 1.5 across the 5 surveys. The prevalence by prefecture showed an increase from 1982 to 2002, but a decrease in all 11 prefectures in 2012 and 2022, except for Okinawa (Table S3). By population density, the prevalence in urban areas was 3.9%, 5.2%, 6.5%, 4.7%, and 2.3%, in middle areas 2.8%, 4.4%, 6.6%, 4.6%, and 2.8%, and in rural areas 1.9%, 4.0%, 6.3%, 6.2%, and 3.8% (Fig. S1). Significant differences among the 3 groups were observed in Phase I and Phase II, but not in Phases III, IV, and V.

There was no significant difference in the prevalence of asthma based on domestic heating type in any of the 5 surveys (Table 2). Similarly, there was no significant association between asthma prevalence and carpet use or pet ownership across all surveys from 1992 to 2022. The prevalence of asthma was significantly higher in children with a history of severe respiratory infection by the age of 2 years (7.9% vs 1.6% for no infection, P < 0.001). No significant correlation was found between the prevalence of asthma and air pollution (NOx, sulfur oxide [SOx], SPM) in any of the 5 surveys, although SPM data were unavailable for the 1982 survey (data not shown).

**Table 2:** Prevalence of asthma and wheeze in relation to domestic heating and cooling type, type of infantile diet, medical history and indoor smoking.

Environment and medical history		Asthma (%)				
		1982	1992	2002	2012	2022
Heating system	Clean type	3.14	4.40	6.19	4.70	2.69
	Mixed type	3.27	4.76	6.98	4.56	2.75
	Dirty type	3.14	5.22	6.83	5.28	2.51
Cooling system	None		4.39	6.22	5.64	2.59
	Fan or air conditioner		4.81	6.52	4.71	2.68
	Centralized air conditioner		4.55	7.53	2.74	0.00
	Others		3.54	6.84	4.52	3.52
Infantile diet	Breast feeding		4.88	6.99	4.62	2.76
	Mixed type		4.52	5.88	4.85	2.52
	Cow's milk		4.44	6.36	5.12	3.00
Respiratory infection	Yes	11.42	12.23	16.35	12.91	7.87
	None	2.40	3.67	4.77	3.05	1.61
	Odds ratio (95%CI)	5.24 (4.70–5.84)∗∗∗	3.66 (3.3–4.01)∗∗∗	3.90 (3.57–4.27)∗∗∗	4.63 (4.26–5.22)∗∗∗	5.21 (4.56–6.06)∗∗∗
Asthmatic bronchitis	Yes	11.77	34.23	35.84	24.64	17.97
	None	1.38	0.87	1.44	0.89	0.37
	Odds ratio (95%CI)	9.54 (8.63–10.55)∗∗	59.30 (52.68–66.75)∗∗	35.26 (34.31–42.67)∗∗	36.41 (31.71–41.80)∗∗	59.74 (47.37–75.34)∗∗
Indoor smoking by family	Yes	3.21	4.58	6.49	4.91	2.84
	None	3.00	4.62	6.58	4.64	2.63
	Odds ratio (95%CI)	1.70 (0.97–1.19)	0.99 (0.91–1.08)	0.99 (0.91–1.07)	1.06 (0.95–1.18)	1.08 (0.92–1.28)
Pet ownership	Yes			5.87	4.73	2.81
	None			6.97	4.73	2.63
	Odds ratio (95%CI)			0.83 (0.76–0.91)∗∗	1.00 (0.89–1.11)	1.07 (0.91–1.25)
Cat ownership	Indoor			5.72	5.24	2.47
	Outdoor			6.32	2.73	1.64
	None			6.58	4.72	2.70
	*p*-value^a^				0.174	0.649
Carpet use	Yes			5.16	4.91	2.66
	None			7.04	4.67	2.70
	Odds ratio (95%CI)			0.72 (0.65–0.80)∗∗	1.06 (0.94–1.18)	0.98 (0.84–1.44)

Environment and medical history		Wheeze (%)				
		1982	1992	2002	2012	2022
Heating system	Clean type	3.70	5.01	5.10	4.31	1.88
	Mixed type	4.06	5.49	5.57	5.06	1.96
	Dirty type	4.02	5.86	5.31	4.47	2.41
Cooling system	None		5.14	5.27	3.90	2.22
	Fan or air conditioner		5.37	5.32	4.46	1.90
	Centralized air conditioner		5.46	3.23	2.05	2.27
	Others		5.38	5.43	4.39	1.87
Infantile diet	Breast feeding		5.47	5.45	4.47	1.86
	Mixed type		4.68	4.43	4.30	2.05
	Cow's milk		5.26	5.49	4.67	1.48
Respiratory infection	Yes	7.74	9.09	7.36	7.59	3.92
	None	3.58	4.74	4.92	3.75	1.48
	Odds ratio (95%CI)	2.26 (1.81–2.54)∗∗	2.00 (1.81–2.24)∗∗	1.54 (1.37–1.72)∗∗	2.09 (1.86–2.34)∗∗	2.71 (2.30–3.26)∗∗
Asthmatic bronchitis	Yes	9.66	18.75	15.01	14.71	8.15
	None	2.74	3.51	3.59	2.43	0.98
	Odds ratio (95%CI)	3.79 (3.47–4.15)∗∗	6.34 (5.81–6.92)∗∗	4.74 (4.31–5.22)∗∗	6.93 (6.23–7.71)∗∗	8.92 (7.48–10.64)∗∗
Indoor smoking by family	Yes	4.06	5.24	5.19	4.75	1.97
	None	3.63	5.18	3.36	4.24	1.87
	Odds ratio (95%CI)	1.12 (1.02–1.23)∗	1.01 (0.93–1.10)	1.57 (1.42–1.75)∗∗	1.13 (1.01–1.25)∗	1.06 (0.87–1.29)
Pet ownership	Yes			6.31	4.48	2.07
	None			5.26	4.39	1.83
	Odds ratio (95%CI)			1.21 (1.11–1.33)∗∗	1.02 (0.91–1.14)	1.13 (0.94–1.36)
Cat ownership	Indoor			5.72	3.78	1.79
	Outdoor			5.21	5.12	4.92
	None			5.26	4.44	1.89
	*p*-value^a^				0.387	0.041∗∗
Carpet use	Yes			5.20	4.34	1.88
	None			5.34	4.43	1.90
	Odds ratio (95%CI)			0.97 (0.87–1.09)	0.98 (0.87–1.10)	0.99 (0.83–1.18)

Chi-square test (∗: p < 0.05, ∗∗: p < 0.01, ∗∗∗: p < 0.001))

### Prevalence of wheeze

**Fig. 3:**
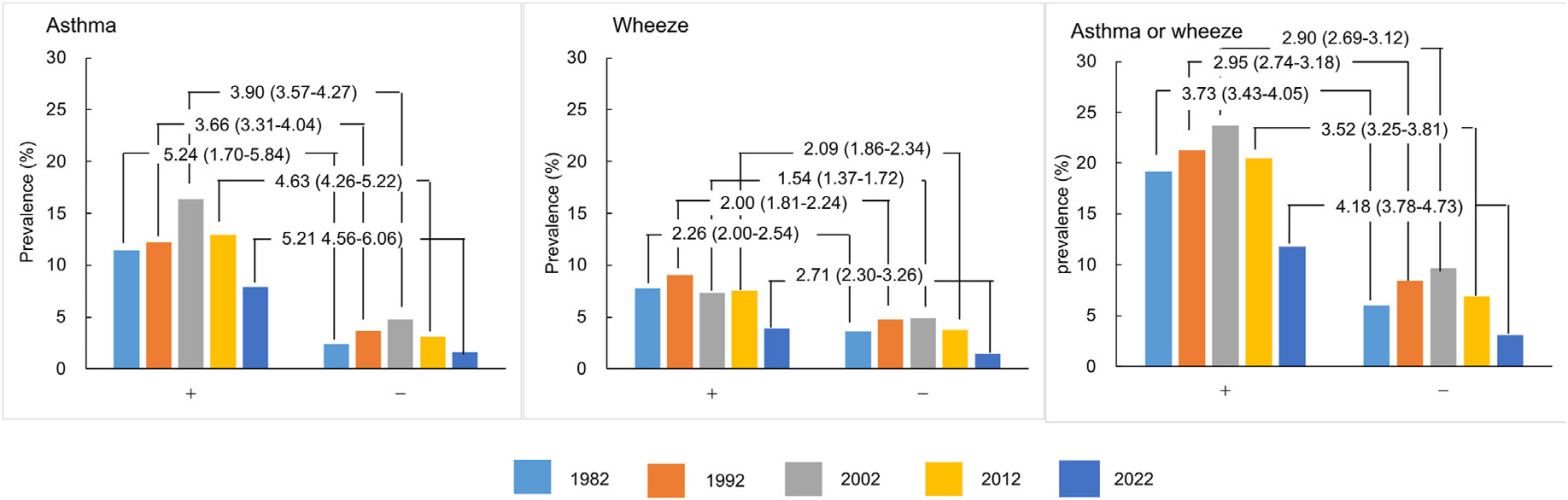
Prevalence rate of asthma and wheeze in relation to frequent respiratory infections under the age of 2. Numeric values in the figures are odds ratio for having asthma, wheezing, or asthma or wheeze in each survey per 10 years using children without frequent respiratory infection under the age of 2 as controls

The prevalence of wheeze followed a similar trend to that of asthma, peaking in Phase III and decreasing in Phases IV and V. By school grade, wheeze prevalence decreased with increasing grade in all phases. The prevalence of wheeze was not associated with domestic heating and cooling systems, infant diet, or indoor smoking in any of the 5 surveys (Table 2). The prevalence of wheeze was significantly higher in children who had had respiratory infections by the age of 2 years than in those who had not (Fig. 3, Table S4).

### Onset and remission of asthma

**Fig. 4:**
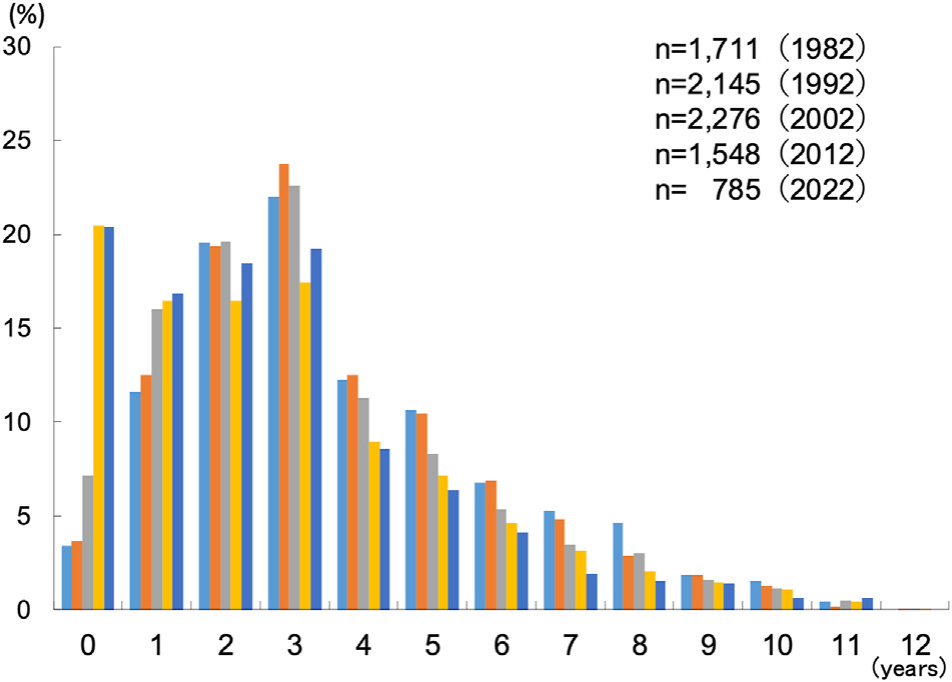
Age at onset of asthma

The age of onset of asthma has been decreasing with each survey, and in the 2022 survey, 74.9% of cases had developed by the age of 3 (Fig. 4). The remission rate of asthma increased from 1.0% in Phase I to 1.6%, 2.4%, 2.6%, and 3.0% in Phases II, III, IV, and V, respectively.

### Comorbid allergic diseases

**Fig. 5:**
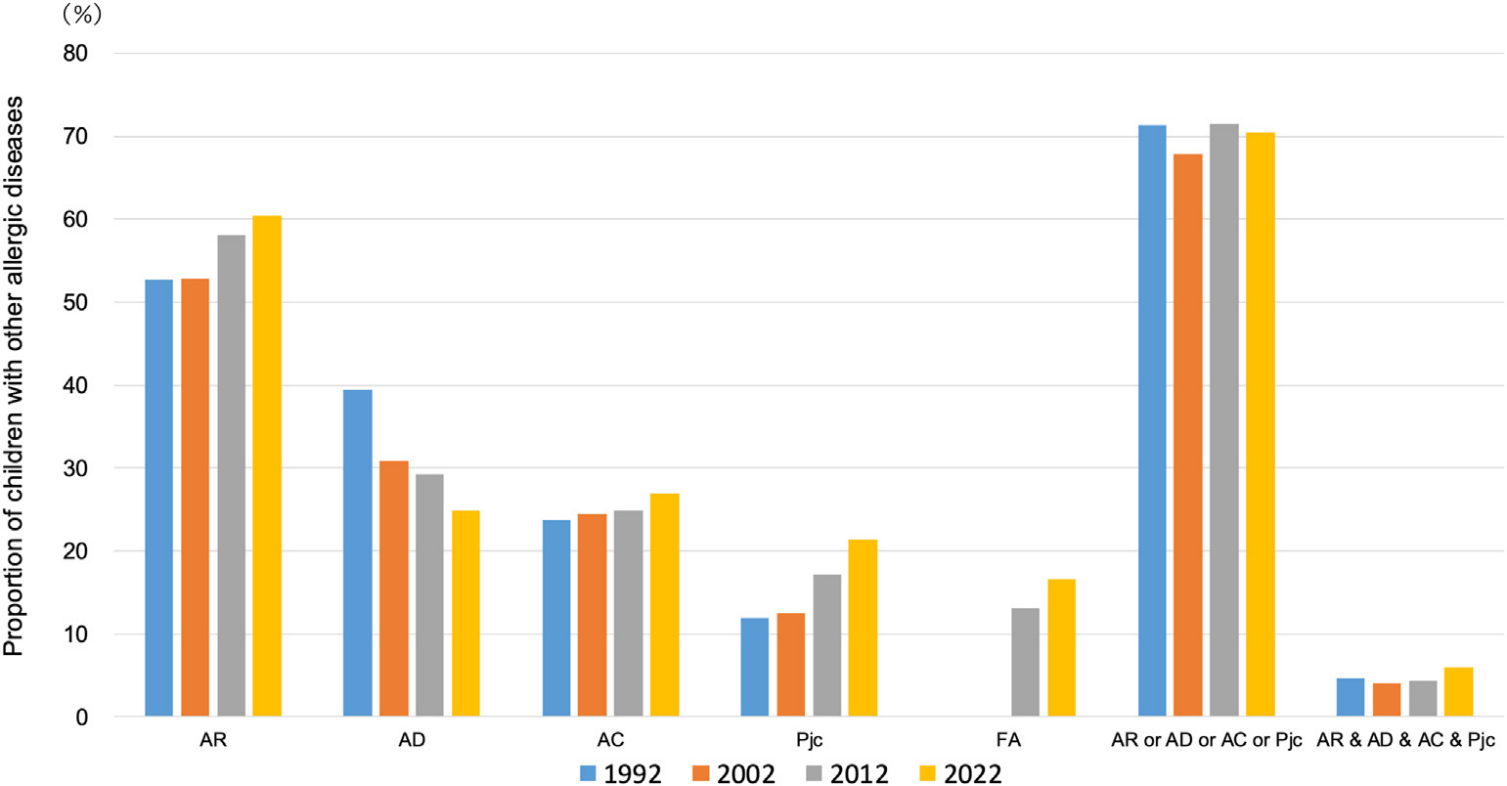
Proportion of children with other allergic diseases among asthmatic children. AD: atopic dermatitis, AR: allergic rhinitis, AC: allergic conjunctivitis, Pjc: Japanese ceder pollinosis

From Phase II onwards, allergic rhinitis was the most common comorbidity, affecting 52.7%–60.4% of children with asthma, followed by allergic conjunctivitis (23.7%–26.9%), atopic dermatitis (24.8%–39.5%), and pollinosis (11.9%–21.4%) (Fig. 5). The proportion of children with any of these comorbidities was 71.3%, 67.9%, 71.5%, and 70.4%, respectively. The proportion of children with all 4 comorbidities was 4.6%, 4.1%, 4.3%, and 6.0%, respectively. Food allergy was first included in Phase IV, with a prevalence of 13.1% in 2012 and 16.5% in 2022. Children with both asthma and food allergy had significantly lower onset ages for asthma than those without food allergy (p < 0.01) (Fig. S2).

### Family history of allergic diseases

The proportion of children with a family history of major allergic diseases among parents and siblings was 41.6% in Phase I, 57.3% in Phase II, 65.5% in Phase III, 69.2% in Phase IV, and 73.8% in Phase V. The prevalence of asthma was 2.6–4.1 times higher in children with a family history of allergic diseases than in those without. The prevalence was even higher for those with a family history of asthma (4.0–5.4 times higher) (Table 3). Baseline characteristics of 5 surveys are shown in Table S5.

**Table 3:** Prevalence of asthma by family history of allergic diseases.

Family History		1982	1992	2002	2012	2022
Major allergy	+	4.82	6.29	8.40	6.11	3.31
	–	1.90	2.23	3.02	1.75	0.84
Odds ratio (95%CI)		2.61 (2.36–2.89)∗∗∗	2.90 (2.61–3.23)∗∗∗	3.04 (2.71–3.42)∗∗∗	4.12 (3.49–4.86)∗∗∗	4.04 (3.12–5.22)∗∗∗
Asthma	+	9.07	11.90	14.25	10.64	6.19
	–	2.44	3.18	4.07	2.37	1.21
Odds ratio (95%CI)		3.99 (3.59–4.44)∗∗∗	4.11 (3.76–4.50)∗∗∗	3.84 (3.53–4.18)∗∗∗	4.94 (4.45–5.50)∗∗∗	5.39 (4.63–6.26)∗∗∗
Eczema	+	5.31	6.86	10.17	7.92	4.02
	–	2.87	3.77	5.26	3.65	2.12
Odds ratio (95%CI)		1.90 (1.68–2.15)∗∗∗	1.87 (1.71–2.05)∗∗∗	2.00 (1.83–2.17)∗∗∗	2.26 (2.04–2.51)∗∗∗	1.93 (1.68–2.22)∗∗∗
Urticaria	+	4.25	7.13	9.28	7.34	3.73
	–	2.87	4.03	5.81	3.95	2.35
Odds ratio (95%CI)		1.50 (1.35–1.67)∗∗∗	1.82 (1.65–2.01)∗∗∗	1.63 (1.48–1.78)∗∗∗	1.95 (1.75–2.17)∗∗∗	1.61 (1.39–1.87)∗∗∗
Allergic rhinitis	+	5.89	6.89	8.79	6.49	3.44
	–	2.61	3.47	4.68	2.79	1.57
Odds ratio (95%CI)		2.34 (2.10–2.59)∗∗∗	2.07 (1.90–2.26)∗∗∗	1.91 (1.75–2.08)∗∗∗	2.40 (2.15–2.68)∗∗∗	2.23 (1.89–2.63)∗∗∗

Chi-square test (∗: p < 0.05, ∗∗: p < 0.01, ∗∗∗: p < 0.001))

## Discussion

The increase in allergic diseases has been a global concern for many years. Despite advances in understanding the pathology of these diseases and improvements in treatment options, their prevalence continues to increase worldwide.

In Japan, the prevalence of asthma in school children in 11 prefectures of western Japan was first surveyed in 1982 (WJSAAC-I), with subsequent surveys in 1992 (WJSAAC-II), 2002 (WJSAAC-III), 2012 (WJSAAC-IV), and 2022 (WJSAAC-V). The prevalence increased from 3.1% in 1982 to 6.5% in 2002, then showed a significant decrease in the subsequent surveys, dropping to 2.7% in 2022.^5^, ^6^, ^7^, ^8^ This decrease was observed consistently across all regions, sexes, and school grades. Further investigation is needed to clarify the reasons behind this trend, particularly the potential impact of significant environmental changes between 2002 and 2022, and especially from 2012 to 2022. The unexpected decline in 2022 suggests that recent changes, possibly related to the COVID-19 pandemic, may have affected asthma prevalence.

During the COVID-19 pandemic, changes in lifestyle and healthcare environments may have reduced respiratory infections in children, thereby decreasing the incidence and exacerbation of asthma symptoms. Reports have shown a significant reduction in asthma-related hospitalizations during the COVID-19 pandemic in Japan, as well as a notable decline in new asthma diagnoses in children during school closures in early 2020.^15^ Similar trends were observed in other countries, where hospital admissions for respiratory diseases such as asthma, bronchiolitis, and pneumonia also decreased significantly.^16^ These findings suggest that measures such as mask-wearing and social distancing may have contributed to the reduced transmission of respiratory infections, which are known triggers for asthma exacerbations.^17^^,^^18^

Another factor to consider is the use of masks, which may have reduced exposure to air pollutants, such as PM2.5, known to exacerbate respiratory symptoms in allergic diseases. The decrease in asthma prevalence observed in our surveys could reflect the combined effects of reduced infection rates and decreased exposure to environmental pollutants.^19^

The definition of asthma used in our surveys followed the America Thoracic Society Division of Lung Disease (ATS-DLD) method, which is stricter than the ISAAC method, leading to lower prevalence, but providing a more accurate diagnosis of asthma. This approach may partly explain the observed trends in asthma prevalence.^20^^,^^21^ Similar decreases in asthma prevalence have been reported in other studies using different methodologies, suggesting a broader trend.^22^^,^^23^

The early onset of asthma has become more common in recent years, with a significant proportion of children developing asthma before the age of 2 years.^24^^,^^25^ This trend was confirmed statistically in our surveys, with the proportion of children developing asthma from ages 6–12 years decreasing over time. These findings support the hypothesis that early childhood factors play a crucial role in the development of asthma. The high incidence of asthma onset before the age of 2 may be influenced by the presence of non-asthmatic causes of recurrent wheezing in infants, such as gastroesophageal reflux disease (GERD) and bronchopulmonary dysplasia (BPD). However, in this study, asthma was defined only in cases where symptoms or asthma treatment had been present within the past 2 years. Therefore, recurrent wheezing caused by conditions other than asthma does not affect the prevalence of asthma reported in this study.

The prevalence of comorbid allergic diseases, such as allergic rhinitis, atopic dermatitis, and allergic conjunctivitis, has also shown significant trends. Whereas allergic rhinitis and conjunctivitis have increased in prevalence, the prevalence of atopic dermatitis has decreased, reflecting changes in environmental and possibly genetic factors. Food allergies, first included in our Phase IV survey, have also shown an increasing trend, with significant differences in asthma onset age between children with and without food allergies.

Family history remains a significant risk factor for asthma, with higher prevalence observed in children with a family history of asthma or other allergic diseases. This highlights the genetic predisposition to asthma and the need for targeted preventive measures in high-risk populations.

The limitations of this study include its cross-sectional design, as it was conducted every 10 years over a 40-year period using the same methodology in the same region; however, it is not a cohort study, meaning that the same population was not continuously followed. Therefore, changes in background factors such as asthma treatment and living environment over the 40-year period may have influenced the results. Additionally, as the study was conducted in a specific region of Japan, the findings may not be fully generalizable to other regions or countries with different environmental and healthcare conditions. The data do not reflect the entirety of Japan. Comparison with national data, such as Japan Environment and Children's Study,^26^ is necessary in the future. Moreover, although the trajectories of wheezing and asthma are well known,^27^ this study did not conduct a detailed longitudinal analysis. Furthermore, potential recall bias in self-reported data and variations in diagnostic criteria over time should be considered when interpreting the results. In addition, this study lacks data on sensitization to inhalant allergens, such as serum-specific IgE. However, a cohort study conducted in another region of Japan (Tokyo) reported that at the age of 9, allergen sensitization was 74.8%, and sensitization to Der f 1 was 54.3%.^28^

In conclusion, the decreasing trend in asthma prevalence observed in our surveys may be attributed to reduced respiratory infections and exposure to environmental pollutants due to recent public health measures. Continued surveillance and analysis are essential to understand the long-term impact of these factors and to develop effective strategies for asthma prevention and management.

## Abbreviations

ISAAC, International Study of Asthma and Allergies in Childhood; WJSAAC, West Japan Study of Asthma and Allergies in Childhood; ATS-DLD, American Thoracic Society - Division of Lung Diseases; NO, Nitrogen oxide; SO, Sulfur oxide; SPM, Suspended Particulate Matter; OR, Odds ratio; COVID-19, CoronaVirus Infectious Disease, emerged in 2019; PM2.5, Particulate Matter 2.5; AD, atopic dermatitis; AR, allergic rhinitis; AC, allergic conjunctivitis; Pjc, Japanese ceder pollinosis.

## Ethics considerations

This study adhered to the principles outlined in the Helsinki Declaration and the ethical guidelines for medical and health research involving human participants, as delineated by the Ministry of Health, Labour, and Welfare of Japan. This study was approved by the ethics committee of Fukuoka Children's Hospital (ID: 2021-963).

## Author's contributions

SN, JT, HO, HM and SH wrote the manuscript. SN, JT, HO, HM and SH designed the study. JT, HO, HM, SH, KO, YG, KK, KT, YK, SY, SA, TK, AS, YS, KT, KF, SS, HY, HM, TN, KN, KH, TF, TF, TM, SM, CT, and ZM collected data, and SH performed statistical analyses and interpreted results. All authors read and approved the final manuscript.

## Author's agreement

All authors agree to the submission and availability of data and materials to WAO.

## Source of funding

This work was supported by a science research grant for research on allergic diseases and immunology from the 10.13039/100009647Ministry of Health, Labour, and Welfare in Japan (grant number: 20FE2001) and the 10.13039/100014423Environmental Restoration and Conservation Agency of Japan.

## Declaration of competing interest

The authors declare they have no conflicts of interest associated with this manuscript.
