# Supplementary material for: Surveys on the prevalence of pediatric asthma in Japan: A comparison between the 1982, 1992, 2002, 2012, and 2022 surveys conducted in the same region using the same methodology (WJSAAC PhaseⅠ∼Ⅴ)
Source: World Allergy Organ J. 2025 Apr 23;18(5):101052. doi: 10.1016/j.waojou.2025.101052 (PMC12051145; doi:10.1016/j.waojou.2025.101052)
Supplement: Multimedia component 1 [file mmc1.pdf]

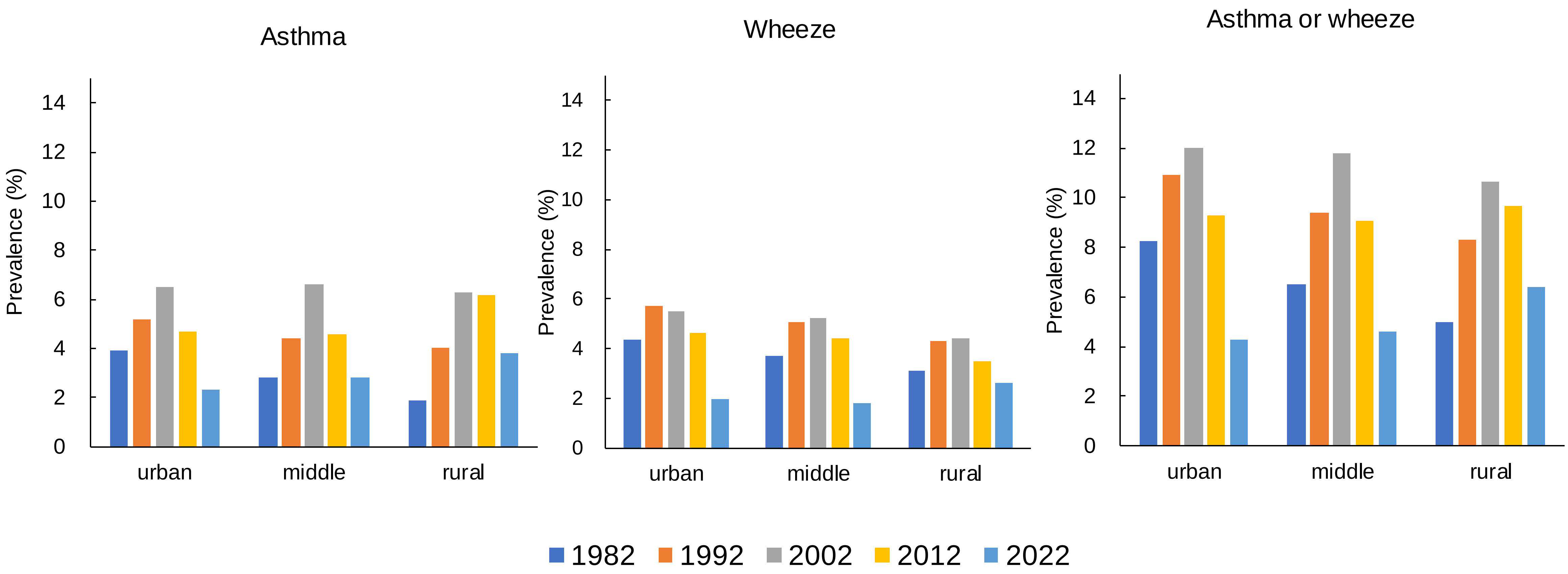

Fig. S1　Prevalence of asthma and wheeze in three areas

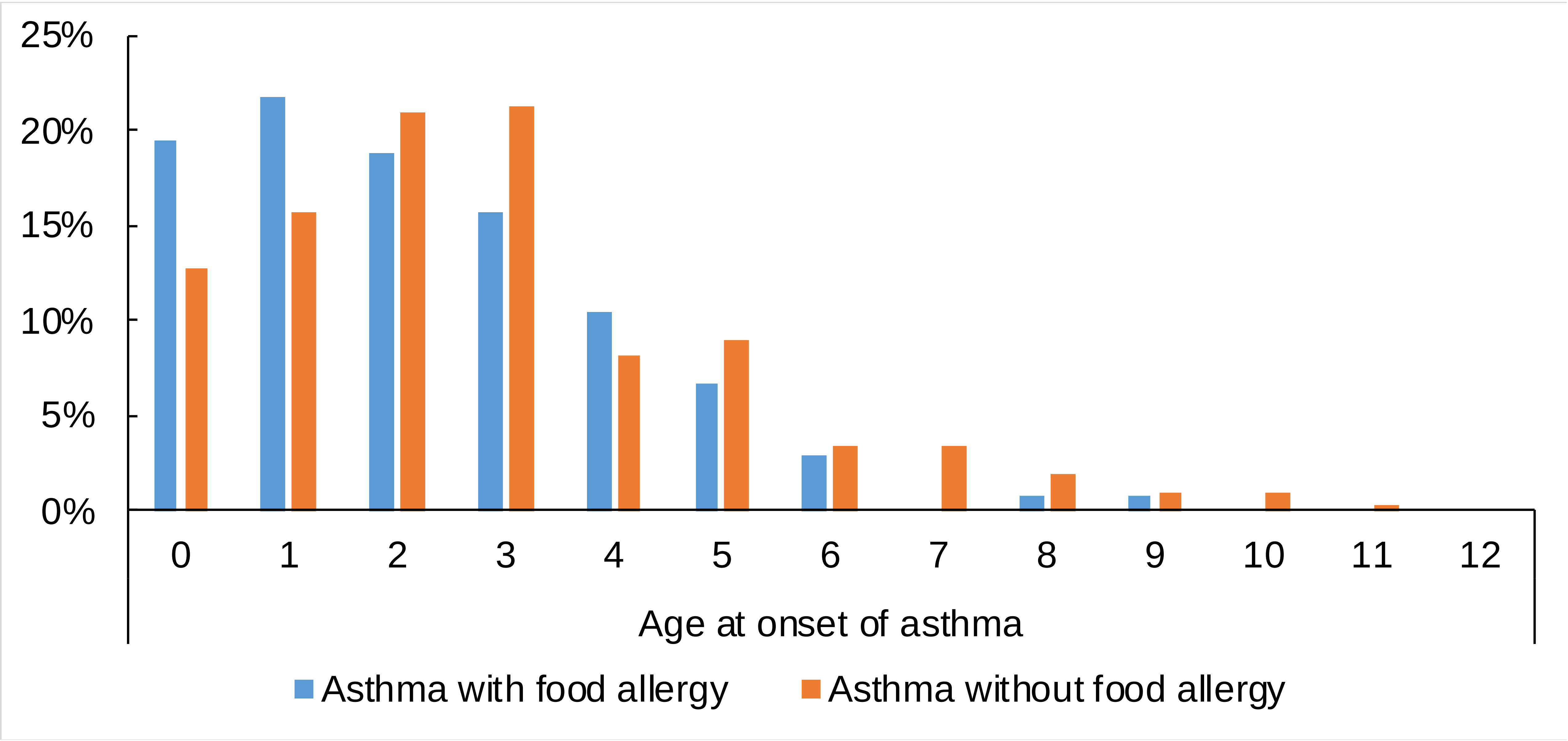

Fig.S2 Age at onset of asthma with or without food allergy
