# Supplementary material for: Surveys on the prevalence of pediatric asthma in Japan: A comparison between the 1982, 1992, 2002, 2012, and 2022 surveys conducted in the same region using the same methodology (WJSAAC PhaseⅠ∼Ⅴ)
Source: World Allergy Organ J. 2025 Apr 23;18(5):101052. doi: 10.1016/j.waojou.2025.101052 (PMC12051145; doi:10.1016/j.waojou.2025.101052)
Supplement: Multimedia component 2 [file mmc2.doc]

Table S1 Prevalence of asthma and wheeze by sex and year at survey

| Diseases | Male | | | | |  | Female | | | | |  | Total | | | | |
| --- | --- | --- | --- | --- | --- | --- | --- | --- | --- | --- | --- | --- | --- | --- | --- | --- | --- |
|  | 1982 | 1992 | 2002 | 2012 | 2022 |  | 1982 | 1992 | 2002 | 2012 | 2022 |  | 1982 | 1992 | 2002 | 2012 | 2022 |
| Asthma (%) | 3.8 | 5.6 | 8.1 | 6.0 | 3.2 |  | 2.5 | 3.6 | 5.0 | 3.5 | 2.1 |  | 3.1 | 4.6 | 6.5 | 4.7 | 2.7 |
| Wheeze (%) | 4.2 | 5.8 | 5.8 | 5.1 | 2.2 |  | 3.6 | 4.6 | 4.7 | 3.7 | 1.6 |  | 3.9 | 5.2 | 5.3 | 4.4 | 1.9 |
| Asthma or wheeze (%) | 8.0 | 11.5 | 11.5 | 11.4 | 5.4 |  | 6.1 | 8.1 | 9.7 | 7.2 | 3.7 |  | 7.1 | 9.8 | 11.8 | 9.1 | 4.6 |

Table S2 Prevalence of asthma and wheeze in 11 prefectures

| Prefecture | Asthma (%) | | | | |  | Wheeze (%) | | | | |  | Asthma or wheeze (%) | | | | |
| --- | --- | --- | --- | --- | --- | --- | --- | --- | --- | --- | --- | --- | --- | --- | --- | --- | --- |
|  | 1982 | 1992 | 2002 | 2012 | 2022 |  | 1982 | 1992 | 2002 | 2012 | 2022 |  | 1982 | 1992 | 2002 | 2012 | 2022 |
| Fukuoka | 3.91 | 5.10 | 6.67 | 4.50 | 2.38 |  | 4.47 | 5.51 | 5.61 | 4.65 | 1.84 |  | 8.39 | 10.60 | 12.28 | 9.15 | 4.21 |
| Saga | 2.48 | 3.85 | 6.28 | 5.03 | 3.11 |  | 3.24 | 4.34 | 4.68 | 3.02 | 2.36 |  | 5.72 | 8.19 | 10.95 | 8.05 | 5.47 |
| Nagasaki | 3.72 | 5.20 | 6.69 | 4.39 | 2.63 |  | 4.19 | 5.36 | 5.32 | 5.12 | 2.08 |  | 7.91 | 10.56 | 12.00 | 9.51 | 4.71 |
| Kumamoto | 3.02 | 4.60 | 6.58 | 4.80 | 2.10 |  | 3.37 | 4.60 | 4.14 | 5.09 | 1.65 |  | 6.39 | 9.20 | 10.72 | 9.89 | 3.75 |
| Kagoshima | 3.20 | 4.58 | 6.82 | 4.43 | 2.82 |  | 4.12 | 6.04 | 5.33 | 4.40 | 2.01 |  | 7.32 | 10.62 | 12.14 | 8.83 | 4.82 |
| Ooita | 2.66 | 4.97 | 6.73 | 4.88 | 3.24 |  | 5.17 | 5.97 | 5.35 | 5.43 | 1.22 |  | 7.83 | 10.94 | 12.08 | 10.31 | 4.46 |
| Miyazaki | 2.97 | 4.33 | 7.65 | 4.82 | 2.59 |  | 2.97 | 4.85 | 6.28 | 4.17 | 1.69 |  | 5.94 | 9.18 | 13.93 | 9.00 | 4.28 |
| Yamaguchi | 2.38 | 4.22 | 8.00 | 5.02 | 2.59 |  | 3.53 | 4.70 | 5.48 | 3.30 | 1.59 |  | 5.91 | 8.92 | 13.48 | 8.33 | 4.18 |
| Okinawa | 2.58 | 3.45 | 4.76 | 6.29 | 3.04 |  | 3.91 | 4.73 | 5.82 | 4.12 | 2.05 |  | 6.49 | 8.18 | 10.58 | 10.41 | 5.09 |
| Hyogo | 2.26 | 3.80 | 5.92 | 5.36 | 3.84 |  | 3.83 | 4.93 | 6.04 | 4.29 | 0.74 |  | 6.09 | 8.73 | 11.96 | 9.65 | 4.58 |
| Kagawa | 2.976 | 4.36 | 4.60 | 3.84 | 2.64 |  | 3.22 | 5.87 | 5.93 | 3.84 | 2.70 |  | 6.19 | 10.23 | 10.53 | 7.69 | 5.34 |
| Mean | 3.14 | 4.60 | 6.54 | 4.72 | 2.68 |  | 3.91 | 5.22 | 5.28 | 4.41 | 1.90 |  | 7.05 | 9.82 | 11.82 | 9.14 | 4.57 |

Table S3. History of frequent respiratory infection under the age of 2 and history of asthmatic bronchitis among children with asthma and those without asthma nor wheeze

|  |  | frequent respiratory infection under the age of 2 | | Asthmatic bronchitis | |
| --- | --- | --- | --- | --- | --- |
|  |  | Yes | None | Yes | None |
| Children with asthma | 1982 | 511 | 1,209 | 1,106 | 632 |
|  |  | 29.7% | 70.3% | 63.6% | 36.4% |
|  | 1992 | 616 | 1,515 | 1,786 | 364 |
|  |  | 28.9% | 71.1% | 83.1% | 16.9% |
|  | 2002 | 895 | 1,459 | 1,924 | 445 |
|  |  | 38.0% | 62.0% | 81.2% | 18.8% |
|  | 2012 | 740 | 856 | 1,398 | 145 |
|  |  | 46.4% | 53.6% | 90.6% | 9.4% |
|  | 2022 | 402 | 401 | 686 | 82 |
|  |  | 50.1% | 49.9% | 89.3% | 10.7% |
| Children without asthma nor wheeze | 1982 | 3,358 | 47,058 | 7,065 | 43,869 |
|  |  | 6.7% | 93.3% | 13.9% | 86.1% |
|  | 1992 | 3,663 | 37,353 | 1,848 | 39,525 |
|  |  | 8.9% | 91.1% | 4.5% | 95.5% |
|  | 2002 | 3,731 | 27,193 | 1,961 | 29,101 |
|  |  | 12.1% | 87.9% | 6.3% | 93.7% |
|  | 2012 | 4,072 | 25,757 | 2,693 | 22,994 |
|  |  | 13.7% | 86.3% | 10.5% | 89.5% |
|  | 2022 | 3,908 | 23,400 | 1,978 | 21,839 |
|  |  | 14.3% | 85.7% | 8.3% | 91.7% |

Table S4. Definition of allergic diseases

| **Allergy Disease** | **Definition** |
| --- | --- |
| **Asthma** | Defined by fulfilling all six of the following criteria: 1. History of wheezing or whistling sounds in the chest accompanied by sudden shortness of breath. 2. Two or more past episodes of such attacks. 3. Diagnosed with asthma, asthmatic bronchitis, or pediatric asthma by a physician. 4. Wheezing sounds were present during the attacks. 5. Shortness of breath accompanied by wheezing during attacks. 6. Symptoms or treatment for asthma, asthmatic bronchitis, or pediatric asthma in the past two years. |
| **Asthma Remission** | Fulfills criteria 1–5 for asthma but does not fulfill criterion 6. |
| **Wheezing** | Meets all three of the following criteria but does not qualify for asthma or asthma remission: 1. Presence of wheezing sounds while breathing. 2. Occurs only when having a cold. 3. Two or more episodes of wheezing in the past two years. |
| **Atopic Dermatitis** | Diagnosed with eczema or atopic dermatitis by a physician and currently has the condition. |
| **Atopic Dermatitis Remission** | Diagnosed with eczema or atopic dermatitis by a physician but does not currently have the condition. |
| **Allergic Rhinitis** | Diagnosed with allergic rhinitis or hay fever-related rhinitis by a physician and currently has either ongoing rhinitis symptoms (sneezing, runny nose, nasal congestion) or experiences worsening symptoms from February to April. |
| **Allergic Rhinitis Remission** | Meets the criteria for allergic rhinitis but does not have current symptoms. |
| **Allergic Conjunctivitis** | Diagnosed with allergic conjunctivitis or hay fever-related conjunctivitis by a physician and currently has either ongoing conjunctivitis symptoms (itchy eyes, redness, excessive tearing) or experiences worsening symptoms from February to April. |
| **Allergic Conjunctivitis Remission** | Meets the criteria for allergic conjunctivitis but does not have current symptoms. |
| **Japanese Ceder Pollinosis** | Meets one of the following two criteria: 1. Diagnosed with pollinosis (hay fever) by a physician and has worsened symptoms from February to April. 2. Diagnosed with allergic rhinitis or conjunctivitis related to hay fever and experiences symptoms that worsen from February to April. |
| **Food Allergy** | Diagnosed with food allergy by a physician and currently has the condition. |

Table S5. Baseline characteristics of five surveys

|  |  |  | 1982 | | | | 1992 | | | | 2002 | | | | 2012 | | | | 2022 | | | |
| --- | --- | --- | --- | --- | --- | --- | --- | --- | --- | --- | --- | --- | --- | --- | --- | --- | --- | --- | --- | --- | --- | --- |
|  |  |  | ｎ | | (％) | | ｎ | | (％) | | ｎ | | (％) | | ｎ | | (％) | | ｎ | | (％) | |
| Total | | | | 55,388 | |  | | 46,718 | |  | | 36,228 | |  | | 33,902 | |  | | 30,024 | |  |
| Sex | | male | 28,036 | | 50.6 | | 23,574 | | 50.5 | | 18,264 | | 50.4 | | 17,217 | | 50.8 | | 15,250 | | 50.8 | |
|  |  | Female | 27,352 | | 49.4 | | 23,144 | | 49.5 | | 17,964 | | 49.6 | | 16,685 | | 49.2 | | 14,657 | | 48.8 | |
| Area | | Urban | 20,421 | | 36.9 | | 14,361 | | 30.7 | | 11,994 | | 33.1 | | 9,960 | | 29.4 | | 9,622 | | 32.0 | |
|  |  | Middle | 31,545 | | 57.0 | | 30,244 | | 64.7 | | 22,706 | | 62.7 | | 22,394 | | 66.1 | | 18,992 | | 63.3 | |
|  |  | Rural | 3,422 | | 6.2 | | 2,113 | | 4.5 | | 1,528 | | 4.2 | | 1,548 | | 4.6 | | 1,410 | | 4.7 | |
| Family  History | Major Allergy | + | 23,069 | | 41.6 | | 26,783 | | 57.3 | | 23,724 | | 65.5 | | 23,126 | | 68.2 | | 22,162 | | 73.8 | |
|  |  | - | 32,319 | | 58.4 | | 19,935 | | 42.7 | | 12,504 | | 34.5 | | 10,776 | | 31.8 | | 7,606 | | 25.3 | |
|  | Asthma | + | 5,952 | | 10.7 | | 7,637 | | 16.3 | | 8,784 | | 24.2 | | 9,777 | | 28.8 | | 8,798 | | 29.3 | |
|  |  | - | 49,436 | | 89.3 | | 39,081 | | 83.7 | | 27,444 | | 75.8 | | 24,125 | | 71.2 | | 20,970 | | 69.8 | |
|  | Eczema | + | 6,453 | | 11.7 | | 12,616 | | 27.0 | | 9,447 | | 26.1 | | 8,550 | | 25.2 | | 8,840 | | 29.4 | |
|  |  | - | 48,935 | | 88.3 | | 34,102 | | 73.0 | | 26,781 | | 73.9 | | 25,352 | | 74.8 | | 20,928 | | 69.7 | |
|  | Urticaria | + | 11,450 | | 20.7 | | 8,623 | | 18.5 | | 7,652 | | 21.1 | | 7,807 | | 23.0 | | 7,187 | | 23.9 | |
|  |  | - | 43,938 | | 79.3 | | 38,095 | | 81.5 | | 28,576 | | 78.9 | | 26,095 | | 77.0 | | 22,581 | | 75.2 | |
|  | Allergic Rhinitis | + | 9,108 | | 16.4 | | 15,504 | | 33.2 | | 16,365 | | 45.2 | | 17,724 | | 52.3 | | 17,691 | | 58.9 | |
|  |  | - | 46,280 | | 83.6 | | 31,214 | | 66.8 | | 19,863 | | 54.8 | | 16,178 | | 47.7 | | 12,077 | | 40.2 | |
| Pet ownership | | Yes |  | |  | |  | |  | | 14,320 | | 39.5 | | 9,519 | | 28.1 | | 7,713 | | 25.7 | |
|  |  | None |  | |  | |  | |  | | 21,908 | | 60.5 | | 24,383 | | 71.9 | | 22,311 | | 74.3 | |
| Cat ownership | | Indoor |  | |  | |  | |  | | 1,520 | | 4.2 | | 1,509 | | 4.5 | | 2,066 | | 6.9 | |
|  |  | Outdoor |  | |  | |  | |  | | 633 | | 1.7 | | 293 | | 0.9 | | 122 | | 0.4 | |
|  |  | None |  | |  | |  | |  | | 34,075 | | 94.1 | | 32,100 | | 94.7 | | 27,836 | | 92.7 | |
| Heating system | | Clean type | 23,767 | | 42.9 | | 21,964 | | 47.0 | | 19,290 | | 53.2 | | 26,047 | | 76.8 | | 26,206 | | 87.3 | |
|  |  | Mixed type | 20,899 | | 37.7 | | 11,975 | | 25.6 | | 11,312 | | 31.2 | | 4,409 | | 13.0 | | 2,549 | | 8.5 | |
|  |  | Dirty type | 10,068 | | 18.2 | | 6,591 | | 14.1 | | 5,626 | | 15.5 | | 2,597 | | 7.7 | | 956 | | 3.2 | |
| Cooling system | | None |  | |  | | 9,041 | | 19.4 | | 2,638 | | 7.3 | | 1,666 | | 4.9 | | 270 | | 0.9 | |
|  |  | Fan or air  conditioner |  | |  | | 26,289 | | 56.3 | | 29,292 | | 80.9 | | 28,783 | | 84.9 | | 28,451 | | 94.8 | |
|  |  | Centralized air  conditioner |  | |  | | 1,209 | | 2.6 | | 186 | | 0.5 | | 146 | | 0.4 | | 88 | | 0.3 | |
|  |  | Others |  | |  | | 3,083 | | 6.6 | | 3,758 | | 10.4 | | 3,031 | | 8.9 | | 908 | | 3.0 | |
